# Long-Term Outcomes After Therapeutic Induction in Patients with Functional Dyspepsia

**DOI:** 10.3390/jcm14228224

**Published:** 2025-11-20

**Authors:** Takayuki Kitano, Toshihiko Tomita, Masatoshi Mieno, Hirofumi Onishi, Hideki Yoneda, Hiroo Sei, Hirotsugu Eda, Toshiyuki Sato, Mikio Kawai, Yoko Yokoyama, Takuya Okugawa, Hirokazu Fukui, Hiroto Miwa, Shinichiro Shinzaki

**Affiliations:** Department of Gastroenterology, Faculty of Medicine, Hyogo Medical University, Nishinomiya 663-8501, Hyogo, Japan; ktakayuki02119@yahoo.co.jp (T.K.); tomita@hyo-med.ac.jp (T.T.); ds23004@hyo-med.ac.jp (M.M.); hi-oonishi@hyo-med.ac.jp (H.O.); hi-yoneda@hyo-med.ac.jp (H.Y.); h-sei@hyo-med.ac.jp (H.S.); eda@hyo-med.ac.jp (H.E.); tshnngn@hyo-med.ac.jp (T.S.); mkawai@hyo-med.ac.jp (M.K.); yoko0502@hyo-med.ac.jp (Y.Y.); okugawat@hyo-med.ac.jp (T.O.); hfukui@hyo-med.ac.jp (H.F.); miwahgi@hyo-med.ac.jp (H.M.)

**Keywords:** functional dyspepsia, long-term outcome, depression, abdominal pain, treatment resistance

## Abstract

**Background**: This multicenter retrospective study evaluated the long-term outcomes of functional dyspepsia (FD) and factors associated with treatment resistance over a 3-year period after treatment initiation. **Methods**: A total of 111 patients diagnosed with FD according to the Rome IV criteria and confirmed by upper endoscopy to have no organic disease were retrospectively analyzed. First-line therapy included acid-suppressive drugs, prokinetics, and the Kampo medicine rikkunshito, whereas second-line therapy comprised anxiolytics or antidepressants for refractory cases. Gastrointestinal symptoms, psychological distress, and health-related quality of life were assessed using the Gastrointestinal Symptom Rating Scale (GSRS), Hospital Anxiety and Depression Scale (HADS), and Short Form-8. Symptom improvement was evaluated at 1 and 3 years. **Results**: The improvement rates at 1 and 3 years were 50.5% and 80.2%, respectively. At baseline, the non-improvement group had significantly higher HADS-Depression and GSRS-Abdominal Pain scores (*p* < 0.05). Multivariate analysis identified HADS-Depression (odds ratio [OR] = 0.676, *p* = 0.019) and GSRS-Abdominal Pain (OR = 0.461, *p* = 0.038) as independent predictors of treatment resistance. **Conclusions**: The present findings indicate that FD often requires prolonged therapy, and baseline depressive symptoms and abdominal pain predict poor long-term outcomes, emphasizing the need for early psychological assessment and integrated management.

## 1. Introduction

Functional dyspepsia (FD) is a condition characterized by upper abdominal symptoms such as stomach pain and bloating, without any underlying organic disease [1,2]. According to the Rome IV criteria, the brain and gastrointestinal tract interact closely, and the clinical symptoms of patients with FD are influenced by environmental and social factors such as life stress and psychological factors such as anxiety, depression, and somatization [3,4,5,6,7,8,9,10]. These factors are collectively referred to as disorders of gut–brain interaction (DGBI) [11,12].

It has been reported that psychological factors affect upper abdominal symptoms, health-related quality of life (HR-QOL), and work productivity in patients with FD [13,14,15,16,17]. Therefore, the importance of considering these factors in diagnostic and therapeutic strategies has been increasingly recognized.

However, current Japanese guidelines from the Japanese Society of Gastroenterology recommend acid-suppressive drugs, prokinetic agents, and Kampo medicine such as rikkunshito as first-line therapies, with no clear treatment guideline based on psychosocial factors [18]. Furthermore, there is a lack of detailed studies examining the long-term course of FD and the impact of pretreatment HR-QOL and psychological factors on treatment outcomes and long-term prognosis. Therefore, it is crucial to develop comprehensive treatment strategies for FD that include psychological factors and accumulate supporting evidence.

In the present study, we aimed to clarify the treatment effects and long-term outcomes in patients with FD after therapeutic induction. We evaluated gastrointestinal symptoms, anxiety and depression scores, and HR-QOL using self-administered questionnaires conducted on patients who presented to the hospital with dyspeptic symptoms such as epigastric pain and bloating and had been diagnosed with FD according to the Rome IV criteria. We then analyzed clinical data over the subsequent 3 years to evaluate the impact of pretreatment gastrointestinal symptoms, anxiety and depression scores, and HR-QOL on treatment outcomes as well as the clinical characteristics of the treatment-resistant patients.

## 2. Materials and Methods

### 2.1. Study Design

This multicenter, retrospective observational study was conducted jointly at our hospital and affiliated institutions (eight general hospitals) between April 2020 and April 2021, and involved 196 patients who presented with dyspeptic symptoms. Among these patients, 111 with FD who completed self-administered questionnaires before treatment and were followed up for a 3-year clinical course were included in the study.

Clinical data, including age, gender, performance status (PS), height, weight, body mass index (BMI), and digestive symptoms, were collected for more than 3 years. Based on these data, the patients were divided into two groups at 1 and 3 years after their initial visit: a group showing significant symptom improvement (improvement group) and a group showing insufficient symptom improvement (non-improvement group). A comparative analysis was then conducted between these groups. Furthermore, patient characteristics and factors at the initial visit that may contribute to treatment resistance were examined.

The study protocol was approved by the Ethics Review Board of Hyogo Medical University (Approval No. 4753). In accordance with the approved protocol for a retrospective study using anonymized existing clinical information, the requirement for individual written informed consent was waived, and an opt-out consent process was implemented. This study was conducted in accordance with the principles of the Declaration of Helsinki. All authors had full access to the study data and approved the final version of the manuscript.

### 2.2. Patients

The inclusion criteria for the present study were: (1) outpatient patients aged 20–75 years; (2) patients with upper abdominal gastrointestinal symptoms, such as postprandial bloating, early satiety, epigastric pain, and heartburn (symptoms were required to be present for at least 6 months before diagnosis and to have persisted during the most recent 3 months); (3) patients who underwent upper gastrointestinal endoscopy within 1 year from the time of their first visit, with no organic diseases (e.g., malignant tumors, peptic ulcers, esophagitis) identified as the cause of the upper abdominal symptoms; (4) patients who were not taking antidepressants, anxiolytics, or antipsychotic medications (the use of gastrointestinal motility agents, acid suppressants, including H2 receptor antagonists, proton pump inhibitors, potassium-competitive acid blockers, gastric mucosal protectants, prostaglandins, and sleep aids was allowed); and (5) patients who were capable of understanding the study purpose and methods and provided written informed consent to participate.

The exclusion criteria were: (1) patients with obvious causes of upper abdominal gastrointestinal symptoms, such as malignant tumors, peptic ulcers, or systemic diseases (e.g., neurological disorders such as Parkinson’s disease, metabolic disorders such as diabetes); (2) patients with a clear cause of symptoms, such as binge drinking, overeating, or significant stress; (3) patients with predominant gastroesophageal reflux disease (GERD); (4) patients with predominant irritable bowel syndrome (IBS); (5) patients with a history of upper gastrointestinal surgery (endoscopic surgery was allowed); (6) patients with concomitant or suspected psychiatric disorders; (7) patients who were considered inappropriate for inclusion in the study for another reason, such as poor PS or a poor general condition; and (8) patients who had undergone *Helicobacter pylori* eradication therapy within the past year or who tested positive for *H. pylori* infection.

### 2.3. Assessments

FD was diagnosed using the Japanese version of the Rome IV diagnostic questionnaire [19]. Patients with dyspeptic symptoms persisting for at least 6 months and continuing over the most recent 3 months were included. Patients who met the diagnostic criteria for IBS were not included. This study primarily involved outpatients, and all included patients were those who were prescribed medications continuously on an outpatient basis. Disease status after treatment was evaluated at 1 and 3 years after the initiation of treatment based on patient-reported outcomes (PROs) assessed in four categories. To assess the exacerbation of FD symptoms, we routinely evaluated upper abdominal symptoms at each outpatient visit. During the 3-year follow-up period, upper gastrointestinal endoscopy was performed every 12–18 months to monitor for any newly developed organic disease, and routine blood tests were conducted at each outpatient visit to assess general clinical status. If symptoms had resolved at the time of consultation, patients were instructed to return for another visit in the event of symptom recurrence. Regarding FD treatment, in accordance with Japanese clinical guidelines, first-line therapy included acid inhibitory drugs, the prokinetic agent acotiamide, and the Japanese Kampo medicine rikkunshito. If these treatments were ineffective after 4–8 weeks, second-line therapy was initiated. Second-line treatment consisted of anxiolytics or antidepressants. Patients were instructed to take medications continuously rather than on an on-demand basis. First-line therapy in responders was maintained for at least 8–12 weeks and continued as long-term maintenance if symptoms remained stable. For patients with persistent or recurrent symptoms despite initial therapy, second-line agents were either added or switched based on clinical judgment. Medication regimens were adjusted during follow-up visits in accordance with symptom fluctuations and patient tolerance.

The evaluation of disease status after therapeutic induction was conducted based on PROs using a four-point Likert-type scale. Treatment efficacy was assessed at outpatient visits 1 and 3 years after the initial consultation. The 1- and 3-year evaluations were conducted within ± 28 days of the respective time points from the initial outpatient visit. Symptom status was categorized into four grades: “good”, “stable”, “unstable”, and “worsened”. “Good” was defined as the complete resolution of gastrointestinal symptoms, such as abdominal pain, “stable” referred to occasional mild symptoms that did not impair daily life, “unstable” indicated intermittent severe symptoms affecting daily activities, and “worsened” was characterized by an increase in symptom severity and frequency, leading to daily life disturbances.

Based on these evaluations, patients were classified into two groups: a symptom improvement group (“good” + “stable”) and a symptom non-improvement group (“unstable” + “worsened”). The primary and secondary outcomes at 1 and 3 years were then analyzed. Symptomatic remission was operationally defined as the complete disappearance of upper abdominal symptoms, such as abdominal pain and bloating, corresponding to a rating of “good” on the four-point scale, that persisted for at least 3 consecutive months. Symptom stability was defined as only occasional, mild upper abdominal symptoms that did not interfere with daily activities, corresponding to a rating of “stable”. Symptom relapse was defined as a reappearance or worsening of dyspeptic symptoms after having achieved remission (“good”) or stability (“stable”), leading to a deterioration of the PRO category to “unstable” or “worsened” and/or the need for an escalation or change in pharmacological treatment. For the main analyses at 1 and 3 years, patients whose symptom status was “good” or “stable” at the respective time point were classified into the symptom improvement group, whereas those rated as “unstable” or “worsened” were classified into the symptom non-improvement group.

The primary outcome, the symptom improvement rate at 1 and 3 years, was evaluated as follows: symptom improvement rate (%) = (symptom improvement)/(symptom non-improvement) × 100. The secondary outcomes included pretreatment evaluations using scores on the Short Form-8 (SF-8), the Hospital Anxiety and Depression Scale (HADS), and the Gastrointestinal Symptom Rating Scale (GSRS). SF-8 assessments were based on the Physical Component Summary (PCS) for physical health and the Mental Component Summary (MCS) for mental health [20,21,22,23,24,25].

To identify factors associated with treatment resistance in FD, a comparative analysis was conducted between the symptom improvement and non-improvement groups using clinical data, including age, sex, PS, height, weight, BMI, and other factors.

### 2.4. Questionnaires (Psychological, HR-QOL, Digestive Symptoms)

Anxiety and depression were assessed using the HADS [20,21,22], which consists of 14 items: seven related to anxiety and seven related to depression. The scores for anxiety and depression were evaluated along with the total score, with higher scores indicating greater psychological distress. HR-QOL was assessed using the SF-8 questionnaire [23,24], which measures eight health concepts: physical functioning, role limitations due to physical health problems, bodily pain, general health, vitality, social functioning, role limitations due to emotional problems, and mental health.

Digestive symptoms were evaluated using the Japanese version of the GSRS questionnaire [25], which is composed of 15 items categorized into five subscales: reflux, abdominal pain, indigestion, diarrhea, and constipation. Each item is rated on a seven-point Likert-type scale, where 1 indicates no impact of gastrointestinal symptoms on daily life, and 7 indicates symptoms that severely impair daily activities. Subscale scores were calculated as the mean of the respective items, and the overall score was derived as the mean of all subscale scores.

### 2.5. Statistical Analysis

All results are expressed as means ± standard deviations. For the comparison between groups, the chi-square test, unpaired t-test, and multivariate logistic regression analyses were performed. For the questionnaire items showing significant differences in the univariate and multivariate logistic regression analysis, receiver operating characteristic (ROC) curves were plotted, and the area under the curve (AUC) and cutoff values were calculated. Based on the cutoff values derived from the ROC analysis, the sensitivity and specificity of predictive factors for the long-term prognosis of FD were determined. Statistical significance was defined as *p* < 0.05 and performed using GraphPad Prism 10, version 10.2.2 (397) (GraphPad Software, LLC, La Jolla, CA, USA) and SPSS Statistics Base 23, version 22 (IBM Corp., Armonk, NY, USA).

## 3. Results

### 3.1. Patient Enrollment

**Figure 1:**
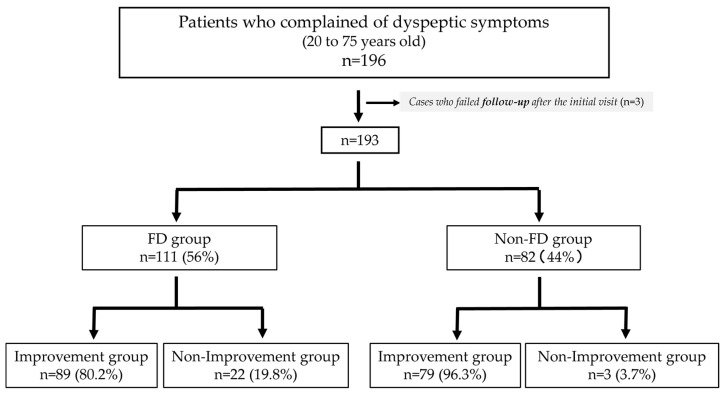
Flowchart showing the patient allocation in the present study. FD, functional dyspepsia.

Among the 196 individuals who visited the outpatient clinic with dyspeptic symptoms, three were excluded because of the difficulty in completing the 3-year follow-up after the initial visit. Three patients did not complete the questionnaire. A total of 193 patients were diagnosed according to the Rome IV criteria, among whom, 111 (56%) met the Rome IV criteria for FD; the other 82 patients (44%) did not meet the criteria and were therefore classified as non-FD (Figure 1). No cases of esophageal or gastric cancer or pancreatobiliary disease were diagnosed during the follow-up period.

The patients’ background characteristics by group are shown in Table 1. No statistically significant differences were found between the FD and non-FD groups in terms of age (*p* = 0.659), proportion of females (*p* = 0.554), BMI (*p* = 0.492), smoking history (*p* = 0.492), alcohol consumption history (*p* = 0.104), or the proportion of patients with a history of *H. pylori* eradication (*p* = 0.836) (Table 1). During the clinical visits, we made every effort to assess the presence of significant stressors or life events. However, no such events were identified at the time of evaluation in this study.

We also compared *H. pylori* infection status and the presence of common comorbidities—including hypertension, dyslipidemia, diabetes, cardiovascular disease, and chronic respiratory disease—between the symptom improvement and symptom non-improvement groups. No significant differences were observed in *H. pylori* infection rates or in the prevalence of any comorbidities at baseline or throughout the follow-up period.

In this study, for first-line treatment, acid-suppressive agents were administered to 105 (94.6%) of 111 patients, acotiamide to 39 patients (35.1%), and the herbal medicine rikkunshito to 10 patients (9.0%). As for second-line treatment, psychotropic agents were used in 36 (32.4%) of 111 patients.

### 3.2. Symptom Improvement Effect and Continuation Rate of Outpatient Visits

**Figure 2:**
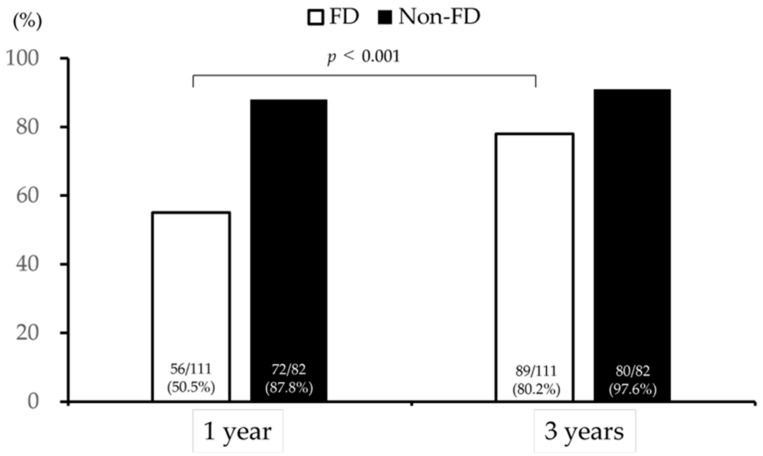
Symptom improvement effect of outpatient visits. FD, functional dyspepsia.

At 1 year after the initial outpatient visit, the symptom improvement rate was 50.5% (56/111) in the FD group and 87.8% (72/82) in the non-FD group. However, at 3 years, the symptom improvement rate increased to 80.2% (89/111) in the FD group and 97.6% (80/82) in the non-FD group (Figure 2). A significant difference in the improvement rate of patients with FD was observed between 1 and 3 years (*p* < 0.001).

Among the 111 patients, 72 had postprandial distress syndrome (PDS), 19 had epigastric pain syndrome (EPS), and 20 had the overlap subtype. The symptom improvement rates at 1 year were 76.4% (55/72) for PDS, 47.4% (9/19) for EPS, and 35% (7/20) for the overlap group. At 3 years, the rates were 81.9% (59/72) for PDS, 84.2% (16/19) for EPS, and 70% (14/20) for the overlap group. Notably, patients with the overlap subtype showed worse symptom improvement.

In the FD group, the continuation rate of outpatient visits was 98.2% (n = 109) at 1 year and decreased to 45.0% (n = 50) at 3 years. This decline in the continuation rate appeared to correspond with the increase in the symptom improvement rate.

### 3.3. HADS Scores of the Two Groups of Patients with FD (Symptom Improvement Group, Symptom Non-Improvement Group)

No significant differences in HADS-Anxiety scores were observed between the symptom improvement and non-improvement groups at either 1 or 3 years (*p* = 0.768, *p* = 0.059, respectively). However, HADS-Depression scores were significantly higher in the symptom non-improvement than in the symptom improvement group at both 1 and 3 years (*p* = 0.025, *p* = 0.004, respectively).

**Figure 3:**
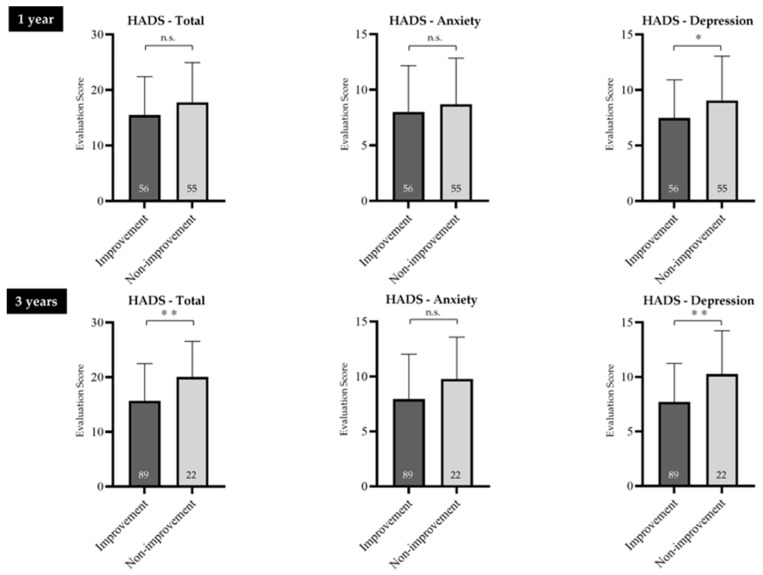
HADS scores of the symptom improvement and symptom non-improvement groups. HADS-Depression scores were significantly higher in the symptom non-improvement than in the symptom improvement group at both 1 and 3 years. FD, functional dyspepsia; HADS, Hospital Anxiety and Depression Scale; n.s., not significant. * Indicates a significant difference (* *p* < 0.05, ** *p* < 0.01) compared with the non-improvement group. The numbers inside the bars represent the numbers of patients. The error bars represent the mean ± standard deviation, visually indicating the variability of each data point.

Regarding the HADS-Total score, no significant difference was noted at 1 year, but at 3 years, significantly higher scores were seen in the symptom non-improvement compared with the symptom improvement group (*p* = 0.008) (Figure 3).

### 3.4. SF-8 Scores of the Two Groups of Patients with FD (Symptom Improvement Group, Symptom Non-Improvement Group)

**Figure 4:**
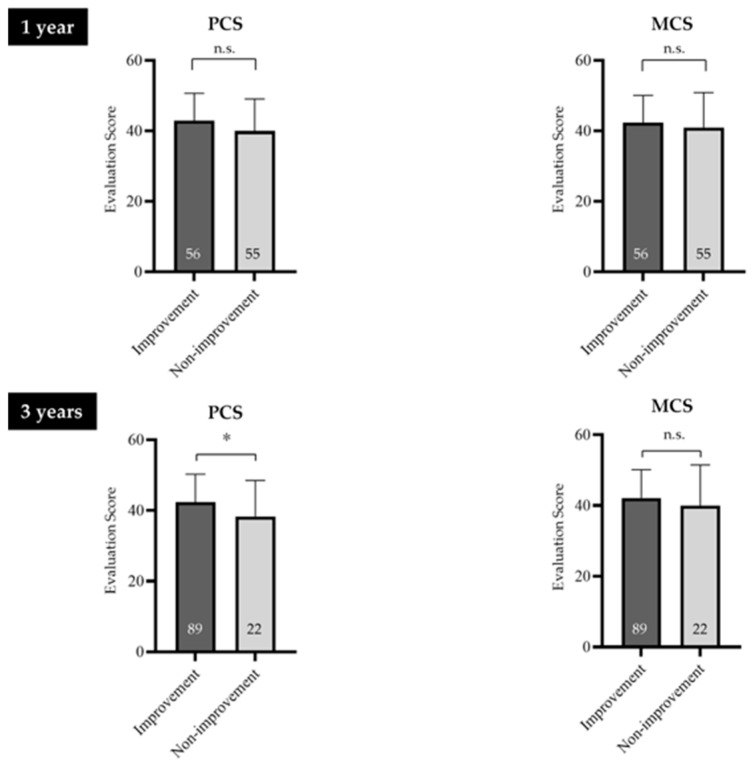
SF-8 scores of the symptom improvement and symptom non-improvement groups. At 3 years, the PCS score in the symptom non-improvement group was significantly lower than that in the symptom improvement group (*p* = 0.045). FD, functional dyspepsia; PCS, Physical Component Summary; MCS, Mental Component Summary; n.s., not significant. * Indicates a significant difference (* *p* < 0.05) compared with the non-improvement group. The numbers inside the bars represent the numbers of patients. The error bars represent the mean ± standard deviation, visually indicating the variability of each data point.

At 1 year after the initial outpatient visit, no significant differences in SF-8 PCS or MCS scores were observed between the symptom improvement and symptom non-improvement groups (*p* = 0.069, *p* = 0.398, respectively). However, at 3 years, the PCS score in the symptom non-improvement group was significantly lower than that in the symptom improvement group (*p* = 0.045) (Figure 4).

### 3.5. GSRS Scores of the Two Groups of Patients with FD (Symptom Improvement Group, Symptom Non-Improvement Group)

At 1 year after the initial outpatient visit, scores on the diarrhea subscale of the GSRS were significantly higher in the symptom improvement compared with the symptom non-improvement group (*p* = 0.045). However, no significant differences in the other subscales (reflux, abdominal pain, indigestion, constipation) or the total score were observed between the two groups (*p* = 0.670, *p* = 0.116, *p* = 0.136, *p* = 0.614, *p* = 0.181, respectively).

**Figure 5:**
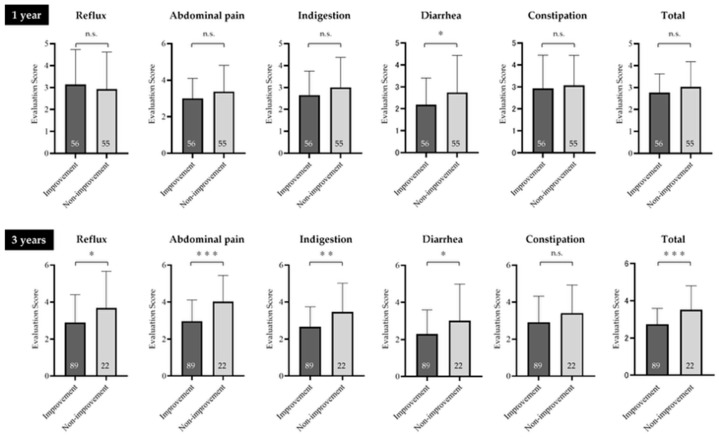
GSRS scores of the symptom improvement and symptom non-improvement groups. At 3 years, the symptom non-improvement group showed significantly higher scores on the reflux, abdominal pain, indigestion, diarrhea, and total score subscales compared with the symptom improvement group. FD, functional dyspepsia; GSRS, Gastrointestinal Symptom Rating Scale; n.s., not significant. * Indicates a significant difference (* *p* < 0.05, ** *p* < 0.01, *** *p* < 0.001) compared with the non-improvement group. The numbers inside the bars represent the numbers of patients. The error bars represent the mean ± standard deviation, visually indicating the variability of each data point.

At 3 years, the symptom non-improvement group showed significantly higher scores on the reflux (*p* = 0.043), abdominal pain (*p* < 0.001), indigestion (*p* = 0.006), diarrhea (*p* = 0.04), and total score (*p* < 0.001) subscales than the symptom improvement group (Figure 5).

### 3.6. Characteristics of Treatment-Resistant Patients with FD (Univariate and Multivariate Analyses)

To identify factors associated with treatment resistance in patients with FD, univariate and multivariate analyses were performed at 3 years. In the univariate analysis, significant differences were observed for seven variables between the symptom improvement and non-improvement groups: HADS-Depression (odds ratio [OR] = 0.826, 95% confidence interval [CI]: 0.721–0.946, *p* = 0.006), HADS-Total (OR = 0.912, 95% CI: 0.85–0.979, *p* = 0.011), GSRS-Reflux (OR = 0.753, 95% CI: 0.569–0.997, *p* = 0.047), GSRS-Abdominal Pain (OR = 0.52, 95% CI: 0.351–0.771, *p* = 0.001), GSRS-Indigestion (OR = 0.605, 95% CI: 0.416–0.879, *p* = 0.008), GSRS-Diarrhea (OR = 0.738, 95% CI: 0.548–0.995, *p* = 0.046), and GSRS-Total (OR = 0.478, 95% CI: 0.297–0.772, *p* = 0.003).

Subsequently, multivariate analysis was conducted on variables that showed significant differences in the univariate analysis. The results indicate that HADS-Depression (odds ratio [OR] = 0.676, 95% confidence interval [CI]: 0.488–0.937, *p* = 0.019) and GSRS-Abdominal Pain scores (OR = 0.461, 95% CI: 0.222–0.959, *p* = 0.038) were significantly associated with treatment resistance (Table 2).

### 3.7. ROC Analysis of HADS-Depression and GSRS-Abdominal Pain Scores in Treatment-Resistant Patients with FD

The usefulness of the HADS-Depression and GSRS-Abdominal Pain scores, which showed significant differences in the multivariate analysis, was evaluated using ROC analysis.

**Figure 6:**
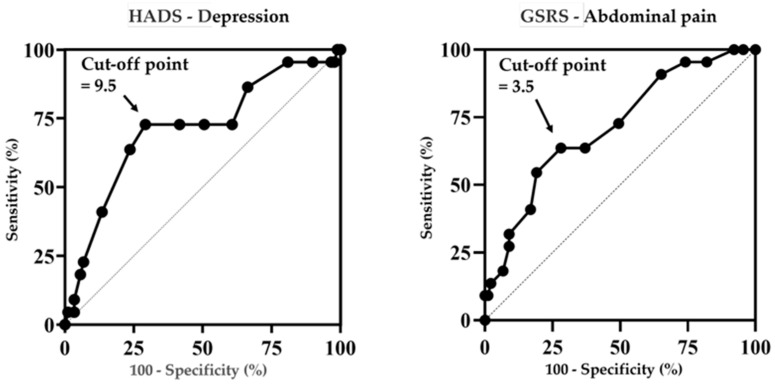
Receiver operating characteristic curve analysis of the HADS-Depression and GSRS-Abdominal Pain scores in treatment-resistant patients with FD. The curve is plotted with sensitivity (*y*-axis) and (100 specificity) (*x*-axis). FD, functional dyspepsia; HADS, Hospital Anxiety and Depression Scale; GSRS, Gastrointestinal Symptom Rating Scale.

Regarding the HADS-Depression score, the cutoff value was 9.5, and the AUC was 0.709 (95% confidence interval [CI]: 0.58–0.84). At this cutoff, the sensitivity was 72.3% and the specificity was 70.8%. Regarding the GSRS-Abdominal Pain score, the cutoff value was 3.5, and the AUC was 0.713 (95% CI: 0.59–0.83). At this cutoff, the sensitivity was 63.6% and the specificity was 71.9% (Figure 6).

## 4. Discussion

This retrospective longitudinal multicenter observational study objectively evaluated the HR-QOL and gastrointestinal symptoms of patients with FD based on the Rome IV criteria. These patients, who presented at outpatient clinics with dyspeptic symptoms such as postprandial fullness and epigastric pain, were assessed using self-administered questionnaires, including the HADS, SF-8, and GSRS. This study aimed to analyze treatment efficacy and identify the characteristics of refractory cases in detail.

The results indicate that the improvement rate for dyspeptic symptoms was 87.8% at 1 year and 97.6% at 3 years in patients without FD, compared with 55.0% at 1 year and 80.2% at 3 years in patients with FD. In particular, a significant difference in the improvement rate of patients with FD was observed between 1 year and 3 years. These findings indicate that treatment for patients with FD requires a relatively longer duration. Additionally, approximately 20% of the patients exhibited poor symptom improvement, even after 3 years, highlighting the presence of refractory cases within the FD population.

Further examination of the characteristics of refractory cases revealed that a high depression score at the initiation of treatment, as well as severe abdominal pain symptoms, was associated with a higher likelihood of treatment resistance.

It is widely recognized from various studies that patients with functional gastrointestinal disorders such as FD and IBS often exhibit a strong placebo effect [26]. This makes it challenging for practitioners in real-world clinical settings to make accurate evaluations of the true therapeutic effects in patients with FD. To address this issue, the present study objectively and scientifically assessed gastrointestinal symptoms and HR-QOL in patients across eight institutions using PROs prior to therapeutic intervention [18]. This multicenter approach allowed for the evaluation of patient conditions while minimizing various biases, thereby enhancing the reliability of the results. These findings hold significant value and underscore the importance of this study.

To date, few reports have been published on the long-term clinical course of patients with FD, and the long-term prognosis for individual patients remains unclear. A single longitudinal, cross-sectional study from Belgium reported that approximately half of patients with FD experienced symptom improvement or resolution over a 5-year follow-up period [27]. In contrast, the present study found that the symptom improvement rates among patients with FD diagnosed using the Rome IV criteria were approximately 60% after 1 year and 80% after 3 years. Among dyspeptic patients who did not meet the Rome IV criteria, a 90% symptom improvement rate was observed after 1 year. The discrepancy between the findings of the Belgian study and the present study could be attributed to several factors. First, the Belgian study defined symptom improvement strictly as “complete resolution or marked improvement”, and due to its cross-sectional design, it may have included cases without continuous treatment or follow-up. In contrast, the present study incorporated a Rome IV-based diagnostic approach and ensured continuous treatment, which likely contributed to the higher symptom improvement rates.

Our previous study demonstrated that patients who did not meet the Rome IV criteria still showed significant impairment in HR-QOL compared with the controls, albeit to a lesser extent than patients with FD [28]. However, the present results confirm that the clinical course of dyspeptic patients who did not meet the Rome IV criteria is favorable. On the other hand, patients with FD required more time for symptom improvement, despite the use of acid-suppressive agents and prokinetic drugs. These findings suggest that the pathophysiology of FD is complex, and that FD is more challenging to treat than dyspeptic conditions not meeting the Rome IV criteria [11,18,29,30,31,32,33,34,35,36,37]. Thus, the results underscore the importance of implementing appropriate therapeutic interventions for patients with FD at an early stage.

Previous studies have reported that the treatment responsiveness of patients with FD is significantly influenced by psychological and social factors [38]. Additionally, the coexistence of psychiatric disorders and nonpsychiatric conditions, such as GERD and IBS, has been shown to have a critical impact on the long-term management of FD. These comorbidities can complicate symptoms and potentially worsen overall treatment outcomes [39]. However, previous research has rarely examined in detail the extent to which baseline gastrointestinal symptoms and psychological status influence treatment outcomes, leaving this relationship unclear. To address this gap, the present study conducted a multivariate analysis at a 3-year follow-up to identify the characteristics of treatment-resistant patients with FD. The results indicate that patients with higher depression scores and more severe abdominal pain at baseline were associated with poorer treatment outcomes. This finding suggests that patients with elevated depression scores or severe abdominal pain before the initiation of treatment require timely and appropriate therapeutic interventions.

The present findings reaffirm the importance of considering mental health in the management of patients with FD. Therefore, future treatment strategies for FD should include not only the assessment of gastrointestinal symptoms, but also the integration of psychological evaluations. Addressing both aspects is likely to play a crucial role in achieving successful treatment outcomes.

Additionally, in this study, we examined the HADS-Depression and GSRS-Abdominal Pain scores in treatment-resistant patients with FD using ROC curve analysis. The results showed that the cutoff values for HADS-Depression and GSRS-Abdominal Pain scores were 9.5 and 3.5, respectively. Furthermore, the HADS-Depression score demonstrated a sensitivity of 72.3% and specificity of 70.8%, while the GSRS-Abdominal Pain score showed a sensitivity of 63.6% and specificity of 71.9%, indicating balanced values for both scores. These results suggest that when managing patients with dyspepsia, evaluating psychological aspects and abdominal pain levels could help predict treatment-resistant cases to some extent. However, the AUC was approximately 0.7, indicating that relying solely on these scores as standalone diagnostic tools may have limitations. Moving forward, combining these scores with other diagnostic tools will be essential in developing appropriate and precise treatment strategies for FD.

This study has several limitations. First, the average age of the enrolled patients was approximately 60 years, which is relatively old. It has been widely reported that FD is more common in younger women [18]; this age difference may have influenced the present findings. Future investigations should include younger patients to provide a more comprehensive understanding of clinical outcomes. Second, although this was a multicenter study, most of the data were collected from tertiary medical institutions, particularly university hospitals, and many patients were referred from other facilities. This might have introduced a selection bias in patient enrollment. However, no significant differences in treatment response rates were observed across facilities. Third, among the patients seen at the hospitals included in this study, approximately 60% met the Rome IV criteria for FD, meaning that the final sample size of enrolled patients was relatively small. In addition, the symptom improvement rate for FD was 50.5% at 1 year and 80.2% at 3 years. Therefore, the number of patients in the non-improvement group at 3 years was relatively small, with only 22 cases. This limitation in the sample size may have influenced the present results.

Another limitation is related to the scoring system used in this study. Each item was uniformly assigned 1 point, although the actual contribution of each factor to treatment resistance may differ. Because the scoring system was exploratory in nature, differential weighting among items was not evaluated. Future studies should aim to develop a more refined and validated scoring model. In the present study, a 3-year follow-up period was adopted in order to reflect real-world clinical practice in Japan and ensure an adequate number of analyzable cases based on previous longitudinal reports, many of which used 2–3 years of follow-up. Although a longer observation period of 5 years or more would be ideal for evaluating the long-term natural history of FD, extending the follow-up beyond 3 years would have substantially reduced the number of cases. Therefore, we defined 3 years as the long-term observation period in this study. Nevertheless, future prospective investigations with longer follow-up durations (≥5 years) are warranted to further clarify the long-term clinical course of FD. In addition, the long-term clinical course of FD may have been influenced by lifestyle and psychosocial changes during the COVID-19 pandemic, such as decreased physical activity, prolonged remote work, and major lifestyle shifts—factors known to worsen FD and IBS symptoms [40]. Because this was a retrospective study, these factors could not be fully assessed, but their potential impact cannot be excluded. Understanding these influences will be important for clinical management in the post-pandemic era.

This study was a multicenter collaborative investigation of the long-term clinical course of patients with FD. The results demonstrate that patients with FD required more time for the improvement of dyspeptic symptoms than the patients without FD. Additionally, patients with higher pretreatment HADS scores, particularly elevated depression scores, or severe abdominal pain, were found to have poorer symptom improvement following treatment. According to the current Japanese guidelines, the first-line treatments for FD are acid suppressants and acotiamide, while psychotropic medications are recommended only as second-line or later options. As a result, in cases where the initial treatment is ineffective, there tends to be a delay in achieving symptom improvement due to the time required for treatment escalation. Therefore, pretreatment assessment of abdominal pain and depressive severity, along with timely consultation with specialists, may be highly effective in achieving early treatment response and symptom relief in patients. Further studies with larger sample sizes are warranted to expand on these findings.

## Figures and Tables

**Table 1:** Characteristics and symptoms of the patients in the present study.

Characteristics and Symptoms	FD Group	Non-FD Group	*p*-Value
Patients (n)	111	82	
Age (years), mean ± SD	60.5 ± 15.4	59.6 ± 13.8	0.659
Gender (n (% female))	71 (64)	49 (60)	0.554
BMI (kg/m^2^), mean ± SD	21.5 ± 4.03	21.9 ± 3.33	0.492
Smoking (n (%))	16 (14.4)	16 (19.5)	0.492
Drinking (n (%))	19 (17.1)	22 (26.8)	0.104
After eradication of *H.p.* (n (%))	39 (35.1)	30 (36.6)	0.836

FD, functional dyspepsia; BMI, body mass index. *H.p.*, *Helicobacter pylori*; SD, standard deviation.

**Table 2:** Univariate and multivariate analyses for poor prognosis in patients with FD.

	Univariate Analysis		Multivariate Analysis	
	Odds Ratio (95% Confidence Interval)	*p*-Value	Odds Ratio (95% Confidence Interval)	*p*-Value
HADS (Anxiety)	0.897 (0.799–1.006)	0.064		
HADS (Depression)	0.826 (0.721–0.946)	0.006	0.676 (0.488–0.937)	0.019
HADS (Total)	0.912 (0.85–0.979)	0.011	1.135 (0.953–1.353)	0.156
SF-8 (PCS)	1.055 (1.00–1.114)	0.051		
SF-8 (MCS)	1.028 (0.976–1.083)	0.299		
GSRS (Reflux)	0.753 (0.569–0.997)	0.047	0.883 (0.492–1.585)	0.677
GSRS (Abdominal pain)	0.52 (0.351–0.771)	0.001	0.461 (0.222–0.959)	0.038
GSRS (Indigestion)	0.605 (0.416–0.879)	0.008	0.651 (0.289–1.465)	0.300
GSRS (Diarrhea)	0.738 (0.548–0.995)	0.046	0.901 (0.498–1.630)	0.730
GSRS (Constipation)	0.792 (0.576–1.008)	0.150		
GSRS (total)	0.478 (0.297–0.772)	0.003	1.611 (0.208–12.465)	0.648

FD, functional dyspepsia; HADS, Hospital Anxiety and Depression Scale; SF-8, 8-Item Short Form health survey; GSRS, Gastrointestinal Symptom Rating Scale; PCS, Physical Component Summary; MCS, Mental Component Summary.

## Data Availability

Any data referred to in this work are available on reasonable request.
